# Biodiversity Observations Miner: A web application to unlock primary biodiversity data from published literature

**DOI:** 10.3897/BDJ.7.e28737

**Published:** 2019-01-16

**Authors:** Gabriel Muñoz, W. Daniel Kissling, E. Emiel van Loon

**Affiliations:** 1 NASUA, Biodiversity research and conservation section, Quito, Ecuador NASUA, Biodiversity research and conservation section Quito Ecuador; 2 Faculty of Arts and Science, Department of Biology, Concordia University, Montreal, Canada Faculty of Arts and Science, Department of Biology, Concordia University Montreal Canada; 3 Faculty of Science, Institute for Biodiversity and Ecosystem Dynamics, University of Amsterdam, Amsterdam, Netherlands Faculty of Science, Institute for Biodiversity and Ecosystem Dynamics, University of Amsterdam Amsterdam Netherlands

**Keywords:** biodiversity data, biodiversity knowledge, biotic interactions, data mobilisation, scientific names, text mining, R.

## Abstract

**Background:**

A considerable portion of primary biodiversity data is digitally locked inside published literature which is often stored as pdf files. Large-scale approaches to biodiversity science could benefit from retrieving this information and making it digitally accessible and machine-readable. Nonetheless, the amount and diversity of digitally published literature pose many challenges for knowledge discovery and retrieval. Text mining has been extensively used for data discovery tasks in large quantities of documents. However, text mining approaches for knowledge discovery and retrieval have been limited in biodiversity science compared to other disciplines.

**New information:**

Here, we present a novel, open source text mining tool, the **Biodiversity Observations Miner (BOM).** This web application, written in R, allows the semi-automated discovery of punctual biodiversity observations (e.g. biotic interactions, functional or behavioural traits and natural history descriptions) associated with the scientific names present inside a corpus of scientific literature. Furthermore, BOM enable users the rapid screening of large quantities of literature based on word co-occurrences that match custom biodiversity dictionaries. This tool aims to increase the digital mobilisation of primary biodiversity data and is freely accessible via GitHub or through a web server.

## Introduction

Mobilisation, digitalization and interoperability of data on biodiversity are vital for sharing our global knowledge of nature (Wilkinson et al. 2016, Kissling et al. 2015, Edwards 2000). The need for digitally available biodiversity data has resulted in the development of global cyber-infrastructures such as the Global Biodiversity Information Facility (GBIF: www.gbif.org) (Edwards 2001), the Plant Trait Database (TRY: www.try-db.org) (Kattge et al. 2011), the Data Observation Network for Earth (DataOne: www.dataone.org) (Michener et al. 2011) and Global Biotic Interactions (GloBi: www.globalbioticinteractions.org) (Poelen et al. 2014). Those efforts have made digital biodiversity data increasingly available in recent years. However, a considerable amount of biodiversity data is still locked inside the current corpus of published literature (Nguyen et al. 2017). This pool of biodiversity data is often stored and shared as PDF files which limits its interoperability. With the increasing availability of literature on the internet, unlocking this biodiversity data and making it digitally interoperable becomes a challenge. Hence, there is a need for developing automatic and semi-automatic computational tools to discover and mobilise biodiversity data contained within this large corpus of literature (Senderov et al. 2017).

Text mining is a computational technique used for the automatic and semi-automatic discovery of useful information from large quantities of text (Hearst 2012). In bio-medicine research, text mining is applied for time-demanding tasks such as document classification and for the discovery of novel potential protein functions and protein-protein interactions (Petrič and Cestnik 2014, Saffer and Burnett 2014, Tari and Patel 2014). Biodiversity data stored within literature can be found in scientific articles (Thessen et al. 2012) or books and monographs (Kissling et al. 2014a). Recently, Algorithms and Application Programmatic Interfaces (APIs) have been developed for the recognition of taxonomic entities and semantic tagging of ecological literature (Nunez-Mir et al. 2016, Pyle 2016, Sautter et al. 2006, Thessen et al. 2012). Furthermore, as ecology moves towards a data-driven science (Michener and Jones 2012), interest in the use of text mining frameworks for data discovery is growing (Miller et al. 2012,Thessen et al. 2012, Thessen and Parr 2014, Nunez-Mir et al. 2016,Senderov et al. 2017, Nguyen et al. 2017,Senderov et al. 2018).

Here, we present the **Biodiversity Observations Miner (BOM)**, a text mining tool that has been designed to augment the ability of ecologists and biodiversity scientists to implement text mining frameworks into their data compilation workflows. A first approach of implementing BOM into biodiversity research is using it as a tool to speed up and standardise the selection of candidate articles for large-scale meta-analyses. In addition, BOM can also be used for rapid discovery of specific biodiversity data across multiple articles at once. As such, this web tool can be used to discover observations from literature and to populate global biodiversity databases, for example on species traits (e.g. TRY) or species interactions (e.g. GloBI). As such, the BOM allows increasing the digital accessibility and availability of biodiversity data. The main feature of BOM is to identify snippets of text that potentially contain biodiversity information (i.e. data of biodiversity observations) within a given corpus of literature. BOM finds these snippets either by finding text statements linked to taxonomic entities (e.g. species names, genus, family) or by using specific keywords to filter a rank of annotated word co-occurrences inside the corpus of literature. These keywords are a curated list of terms describing a particular biodiversity observation and are provided in BOM as biodiversity dictionaries. Biodiversity Observations Miner is open source and freely accessible via GitHub (BiodiversityObservationsMiner) or via a web server (goo.gl/wt6V9R).

## Project description

### Design description

**Figure 1.**
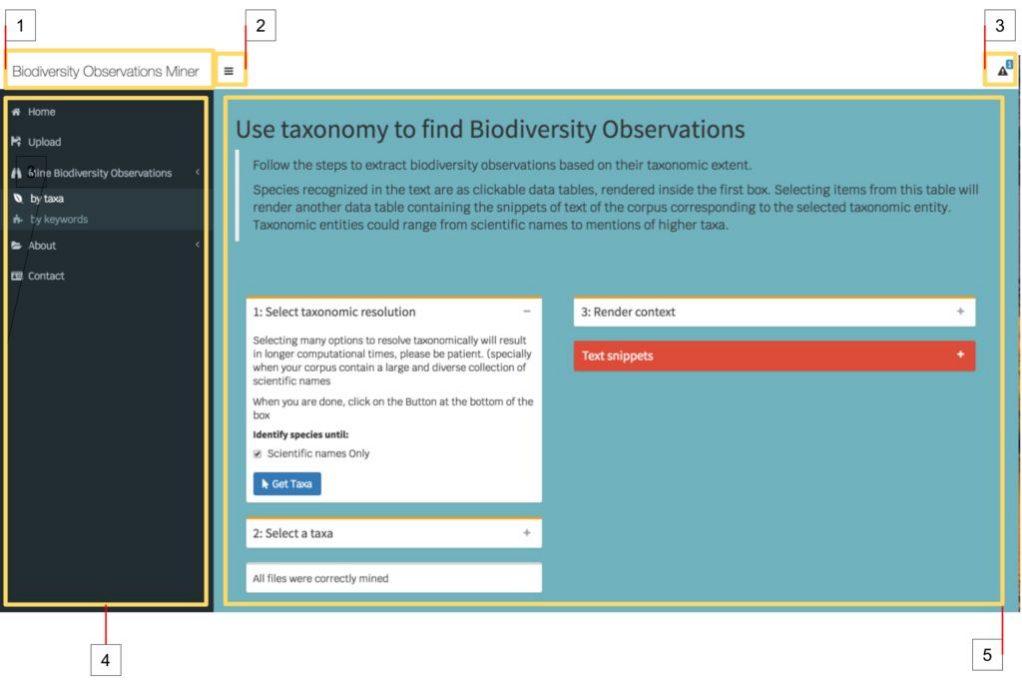
Sections of Biodiversity Observations Miner (BOM) user interface: The figure illustrates the different parts that compose the user interface of BOM web application. The interface is composed of three main components, a header (white bar on top), a sidebar menu (dark blue at in the left side) and the main page (cyan in the centre). The header includes the application name (1), a button to collapse the sidebar menu (2) and a notification menu (3). The sidebar menu (4) contains the individual tabs to navigate across the functionalities of BOM. The main page (5) allows the setting of parameters and obtaining the results of the mining steps. In the main page, the header of setting type boxes are colour-coded yellow whereas the result boxes (i.e. Text snippets) are colour-coded with red headers.

**User interface**:

The web application follows a dashboard design containing a header, a sidebar menu and the main page (Fig. 1). The dashboard header is placed at the top of the screen where users can find the application name (i.e. Biodiversity Observations Miner), a button to collapse the sidebar menu and a notification menu. The sidebar menu is located at the left side and allows easy navigation across all the specific functionalities of BOM. Clicking on each of the tabs in the sidebar menu will render a different content in the main page. The main output of the BOM consists of a list of text snippets, each a sentence long, indexed and annotated across all literature uploaded to the application. Thus, a user can perform a rapid literature search by filtering the output snippets based on taxonomic content (using scientific names present in the text) or biodiversity dictionaries (using curated lists of biodiversity terms). In addition, the application provides an overview of the semantic context of text snippets by calculating patterns of word co-occurrences.

**Functional description**:

OCR of PDF files

Before using Biodiversity Observations Miner, a user needs to create a corpus of relevant literature, stored as a collection of individual PDF files. This biodiversity literature corpus can be compiled by downloading PDFs of scientific articles from web databases such as Web of Science and Google Scholar. The collection of PDF files can be uploaded in batch to BOM. PDF versions from different publications can be very heterogeneous in nature. As such, plain text from PDF file(s) is recognised with the Google Tesseract tool for Optical Character Recognition (OCR) (Smith 2007). The Tesseract tool is a proven, well known, open-source OCR engine which can recognise many languages (Smith 2007). BOM performs the OCR of text with the Tesseract tool using the binding available in the scrapenames function from the taxize package (Chamberlain and Szöcs 2013). However, a portion of PDF files available in web databases does not come in machine-readable format. For example, digitised versions of old papers are usually stored as separate scanned images inside a single PDF file. Currently, BOM cannot handle this type of files and the user will be notified about the presence of such files in the literature corpus within the notification menu (*see* User's manual) (Suppl. material 1). For future updates of BOM, we will seek to include ways to automatically recognise and OCR all type of PDF files, including those with text stored as images.

Scientific name recognition

Biodiversity Observations Miner makes use of the Global Names Recognition and Discovery (GNRD) (Mozzherin et al. 2017) application programme interface (API) to recognise scientific names present of the OCR text. This API is part of the Global Names Architecture (GNA) (Pyle 2016), a name-based cyber-infrastructure which offers a set of open and free web services to find, index and organise biological scientific names (Mozzherin et al. 2017). It includes an algorithm (*biodiversity*) that parses scientific names from text with high accuracy (Mozzherin et al. 2017). Latin words, journal names or terms that resemble the Latin binomial structure of scientific names can cause confusions to the algorithm. However, errors in recognition are usually attributed to false positives rather than false negatives (Mozzherin et al. 2017). A current drawback of the *biodiversity* algorithm is that common names of species are not recognised in the corpus text. BOM includes a search option for taxonomic identification at higher taxonomic ranks (i.e. Family and Class) of the species names recognised in the text. This information is retrieved by querying the National Center of Biotechnology Information (NCBI) taxonomic database using the E-utilities RESTful API of NCBI. Functions to connect to both APIs are implemented in the R package taxize (Chamberlain and Szöcs 2013).

Calculating word co-occurrences

Individual sentences across the whole literature corpus are considered as text snippets that potentially contain one or more biodiversity observations of particular interest for a user of BOM. As such, word co-occurrence patterns can provide useful information to characterise the content of these text snippets. For example, the words "body" + "size" can be used to tag individual text snippets with information on allometric relations, functional trait relationships etc. In BOM, text strings from the literature corpus are split into sentences using a sentence tokeniser. Then, the individual elements (e.g. nouns, verbs, articles) of these sentences are annotated with a pre-trained, English based, natural language processing (NLP) model (Straka and Straková 2017). Finally, a skip-n-gram model is applied to the pool of tokenised sentences.

**Figure 2.**
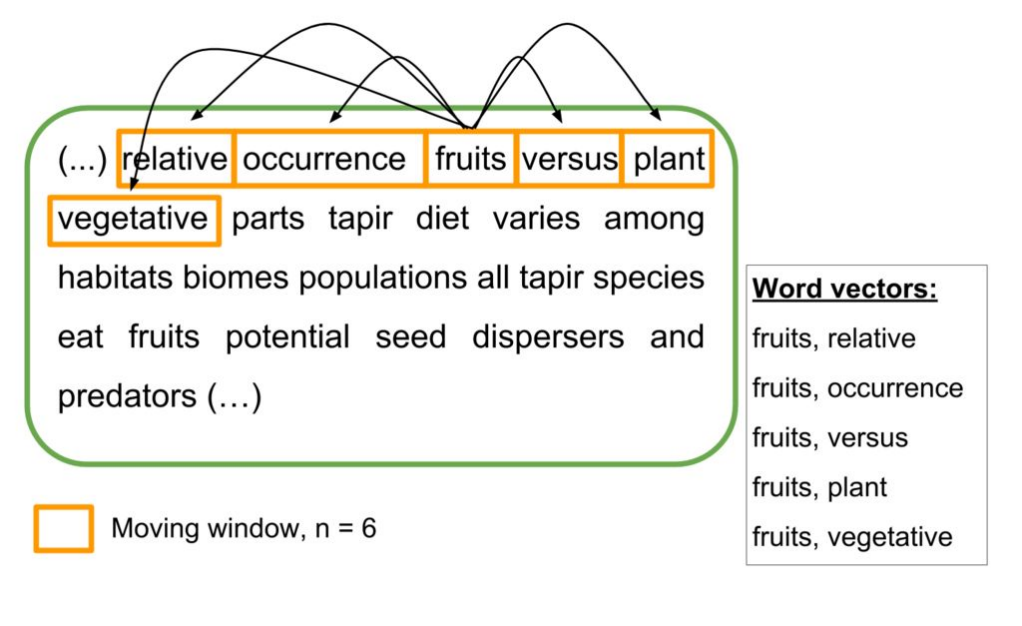
Example of a moving window of n = 6 of a skip-n-gram model over a piece of text from O'Farrill et al. (2013). The text has been cleaned of common stop words (e.g. "the", "all", "however"). Inside the moving window, a central word is fixated (randomly) and all possible word pairs are considered as word vectors. After this step is completed, the moving window advances one word and repeats the process again. Frequencies of co-occurrences within the pool of word vectors are further used to rank word pairs.

The skip-n-gram model is a practical, powerful model to infer context from text and is usually applied in processes such as speech recognition (Silge and Robinson 2016, Thessen et al. 2012). The value of "n" in the model defines the size (i.e. number of words) of the moving window applied to find word vectors in continuous text. These word vectors are constructed by selecting word pairs composed of a fixed word and all other possible combinations of words inside the moving window (Fig. 2). Word pairs are pooled together disregarding the individual distances between the fixed word and the other words inside the moving window. In BOM, a n = 6 was considered to construct the skip-n-gram model and we only included nouns, verbs, and adjectives into the moving window. This was done to prune common stop words (e.g. "the", "all", "and", "however") for co-occurrence calculations. The udpipe (Straka and Straková 2017) package for R was used for sentence tokenization, annotation and to apply the skip-n-gram model. Word co-occurrences are sorted by frequency counts before being presented to BOM users.

Retrieving text snippets

BOM uses indexed scientific names and word co-occurrences to retrieve text snippets across all the uploaded literature corpus. This allows rapid discovery of targeted biodiversity observations inside the corpus text. First, with the **byTaxa** tab, the use of scientific names to retrieve text snippets and word co-occurences to characterise its content allows for rapid screening of literature based on the particular taxonomic interest of an individual user. Second, with the **byKeywords** tab, BOM also allows the retrieval of text snippets based on individual word co-occurrences only. These word co-occurrences can be further filtered using custom biodiversity dictionaries.

Biodiversity dictionaries

**Figure 3.**
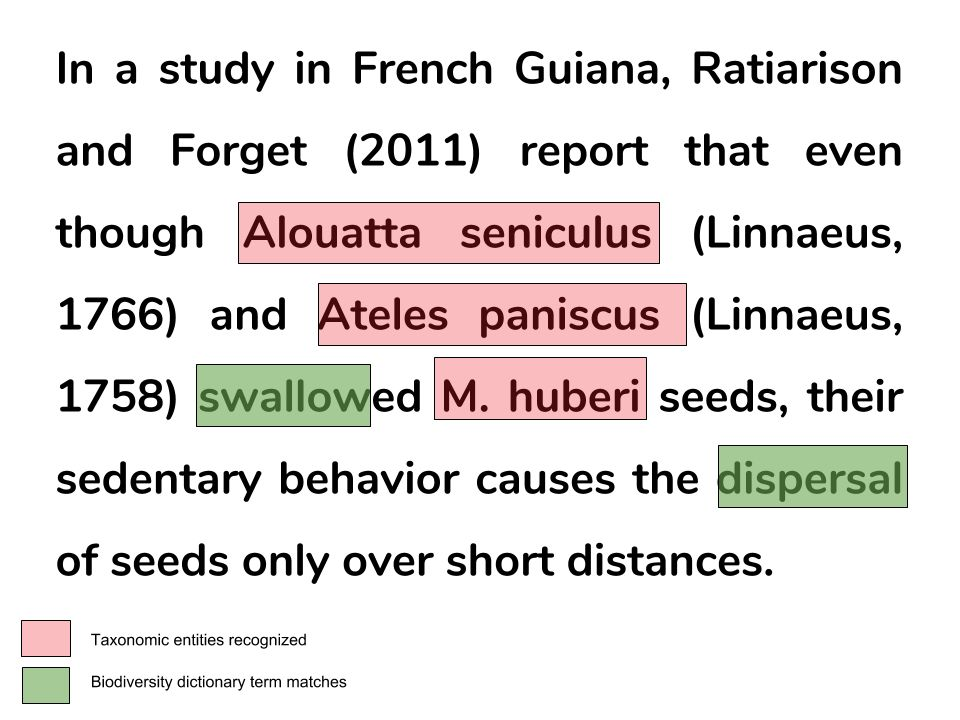
Example of one text snippet resulting from running Biodiversity Observations Miner with O'Farrill et al. (2013) as input. This text snippet (*i.e. biodiversity observation*) contains data about a **frugivory** interaction between plants and animals. Here, biodiversity data comes from the description of the monkeys *Alouatta seniculus* and *Ateles paniscus* being frugivores of *M. huberi* fruits. The terms "swallow" and "dispersal" were part of the frugivory biodiversity dictionary included in BOM. Red boxes highlight the taxonomical entities recognised using the Global Names Architecture API implemented with the taxize (Chamberlain and Szöcs 2013) R package. The green boxes show the matches of frugivory dictionary terms within the text snippet.

A biodiversity dictionary is a list of common terms used to describe a particular biodiversity observation. Currently, BOM lists biodiversity dictionaries matching text observations of frugivory and pollination, i.e. specific biotic interaction types. For example, the written description of a plant-animal interaction of frugivory might include terms such as *fruit*, *eat*, *disperse*, *swallow*, etc. (Fig. 3). Terms included in those biodiversity dictionaries were manually selected from a unigram term-frequency matrix created from sample articles known to contain biodiversity observations on frugivory or pollination. In creating these dictionaries, we limited the length of terms composing the biodiversity dictionary by discussing the rationale behind each term and eliminating ambiguous terms that might match a large number of false positive snippets (i.e. snippets containing non-relevant information). However, because of the intrinsic heterogeneity of natural language to store biodiversity information, certain terms might match other type of observations. However, in BOM, terms in the biodiversity dictionaries are used to optionally filter the list word co-occurrences and not to index text snippets *per se*. This allows the user to finally determine if a particular combination of co-occurring words might lead to snippets containing information of interest (e.g. "eat" + "fruit" = *frugivory* whereas "eat" + "prey" = *predation*). In future updates, we aim to include more biodiversity dictionaries in the web version of BOM. Nevertheless, users running the application locally can also easily integrate custom biodiversity dictionaries of their own (see User's manual: Suppl. material 1).

## Web location (URIs)

Homepage: https://fgabriel1891.github.io/BiodiversityObservationsMiner/

Download page: https://fgabriel1891.github.io/BiodiversityObservationsMiner/

Bug database: https://github.com/fgabriel1891/BiodiversityObservationsMiner/issues/

## Technical specification

Platform: shiny, R.

Programming language: R

Operational system: Windows, OSx, Linux

Interface language: shiny-dashboard, shiny

## Repository

Type: Git

Browse URI: BiodiversityObservationsMiner

## Usage rights

### Use license

Other

### IP rights notes

Creative Commons Attribution 4.0 License. **CC-BY 4.0**

## Implementation

### Implements specification

Published literature in ecology holds a vast amount of information from centuries of research (Miller et al. 2012, Nunez-Mir et al. 2016). However, digitally storing this knowledge as text, in PDF files, limits its openness and accessibility. Thus, as Ecology moves towards a data-driven science (Michener et al. 2011, Petrič and Cestnik 2014, Senderov et al. 2017), the need for easy and standard access to biodiversity data increases (Edwards 2000, Michener and Jones 2012, Kissling et al. 2014, Kissling et al. 2018). Although recent publication practices are increasing the mobility and discoverability of biodiversity data (e.g. Wilkinson et al. 2016), finding information from literature can become challenging and time-consuming. In this sense, Biodiversity Observations Miner is a piece of software which contributes to the discovery, mobilisation and reuse of ecological data stored in scientific literature. BOM can be implemented inside biodiversity research workflows to filter candidate studies in meta-analysis, to discover biodiversity observations for testing hypothesis and to populate global-scale standard biodiversity databases like the Plant Trait Database (TRY: www.try-db.org) (Kattge et al. 2011), the Data Observation Network for Earth (DataOne: www.dataone.org) (Michener et al. 2011) or Global Biotic Interactions (GloBi: www.globalbioticinteractions.org).

In ecology and biodiversity science, computational methods such as machine learning algorithms have slowly integrated into research frameworks when compared with other disciplines (Thessen 2016). Within the field of biodiversity data discovery, recent developments are making substantial progress to bridge this computational gap in ecology (Edwards 2000, Pyle 2016, Garnier et al. 2017, Mozzherin et al. 2017, Senderov et al. 2017, Senderov et al. 2018). As such, the use of proven algorithms through APIs and the open access of digital infrastructures such as the GNRD (Pyle 2016) will certainly foster future open software developments and digital workflows directed towards all research stages in ecology and biodiversity science. Text mining biodiversity observations of species functional traits and biotic interactions is particularly promising and can serve as a starting point to fill knowledge gaps that limit the advancement of ecology and biogeography as a science (Hortal et al. 2015).

The heterogeneity on terminologies describing particular biodiversity observations creates a challenge to automatically characterise text-based observations into standardised biodiversity data. Currently, there is a lack of standard terminologies to describe particular biodiversity observations. For instance, the term "eat" might match the textual description of many forms of biotic interactions (e.g. predation, frugivory, commensalism). We believe that initiatives, such as BOM, can benefit from future work that promotes the standardisation of terms via ontologies and controlled vocabularies. Furthermore, this could be further expanded to increase biodiversity dictionaries to match observations of natural history (e.g. dispersal distances, habitat preferences), biotic interactions (e.g. parasitism) or species functional traits (e.g. leaf area, flower phenology, body mass, wing length, mandible type, lifetime reproductive output) (Cornelissen et al. 2003, Moretti and Legg 2009, Kissling et al. 2018).

### Audience

The target audience for this web application includes ecologists and biodiversity scientists at all career stages. Additionally, this application invites developers (ecologists or not) to suggest ideas for improvement. We are open to discussing additional ideas or new tools to expand the current functionalities of this web application.

## Additional information

### Dependencies

Biodiversity Observations Miner was written in R (R Development Core Team 2015) using the shiny (Chang et al. 2017) R package. Application user interface (UI) was built using the shiny-dashboard R package (Chang and Borges Ribeiro 2018).

Biodiversity Observations Miner makes use of R packages designed for text mining and base R functions. The taxize package is used to establish the API connection to the Global Names Recognition and Discovery (GNRF) tool. Taxize is also used for Optical Character Recognition (OCR) of the text in the PDFs and is done by GNA using the Google Tesseract Tool. The stringr is used for string manipulation. Details on the code and custom functions written for this application can be found in the GitHub Repository of this application. In addition, BOM requires the following R packages to run locally: *shiny*, *shinydashboard*, *stringi*, *stringr, taxize, reshape, udpipe, tibble, DT*.

## Supplementary Material

Supplementary material 1 BOM_USER_MANUAL Data type: user's manual
Brief description: Biodiversity Observation User's manual. Follow this guide to upload literature and mine biodiversity observations using BOM.
File: oo_243479.pdf Gabriel Muñoz
