# Supplementary material for: Biodiversity Observations Miner: A web application to unlock primary biodiversity data from published literature
Source: Biodivers Data J. 2019 Jan 16;(7):e28737. doi: 10.3897/BDJ.7.e28737 (PMC6344444; doi:10.3897/BDJ.7.e28737)
Supplement: Supplementary material 1 — BOM_USER_MANUAL [file bdj-07-e28737-s001.pdf]

Biodiversity Observations Miner: A web application to unlock primary biodiversity data from published literature

Gabriel Muñoz, W. Daniel Kissling & E. Emiel van Loon

USER'S MANUAL

Nov. 2018.

Background:

A large amount of biodiversity information is currently stored in PDF versions of published articles. Biodiversity Observation Miner (BOM) is a web application, written in R, that allow the semi-automatized discovery of punctual biodiversity observations (e.g. biotic interactions, species traits, and natural history descriptions). Furthermore, this tool can be used to screen large quantities of literature based in word co-occurrences matching custom biodiversity dictionaries. his tool aims to increase the digital mobilization of primary biodiversity data and is freely accessible via GitHub or through a web server.

1.- Creating a corpus of literature to mine.

Before starting to use BOM, the user must to compile the collection (corpus) of PDF files that is interested to mine. PDF files with biodiversity literature can be compiled either from downloading PDF articles from a web database (e.g. Web of Science, Google Scholar), with PDf files which a user already owns, or a mixture of both. We recommend placing all the PDF files into a single folder and name each file appropriately (e.g. Farrill_et_al_2006_Tapirs.pdf). Reference management software (e.g. Mendeley, Zotero, EndNote) can be useful to name PDF files in batch and appropriately.

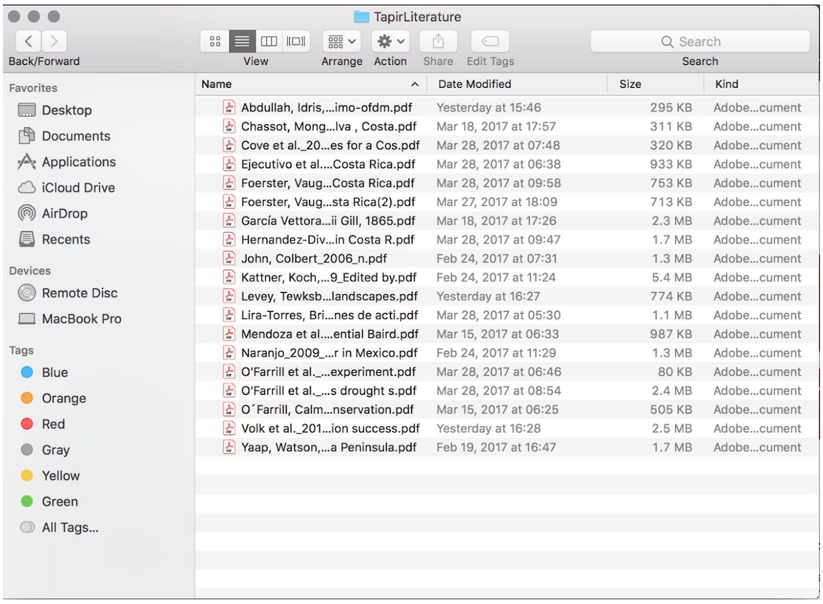

Figure 1: Example of a folder containing PDF files to be mined with BOM. Note that the folder only contains PDF documents and filenames correspond to the format “author(s)_year_title.pdf” of each reference.

2.- Opening Biodiversity Observations Miner

2.1.- Vía web server:

Go to the following link: https://fgabriel1891.shinyapps.io/biodiversityobservationsminer/

Currently the web server has a limitation of use. We recommend the use of the web server only test the functionalities of BOM or to be used in portable devices (e.g. tablets). If you are planning to use it extensively please use the GitHub step.

2.2.- Running BOM locally at your computer

a) Go to BOM GitHub page: https://fgabriel1891.github.io/BiodiversityObservationsMiner/
b) Download the repository files as .zip or .tar. Save it into your computer and decompress it.

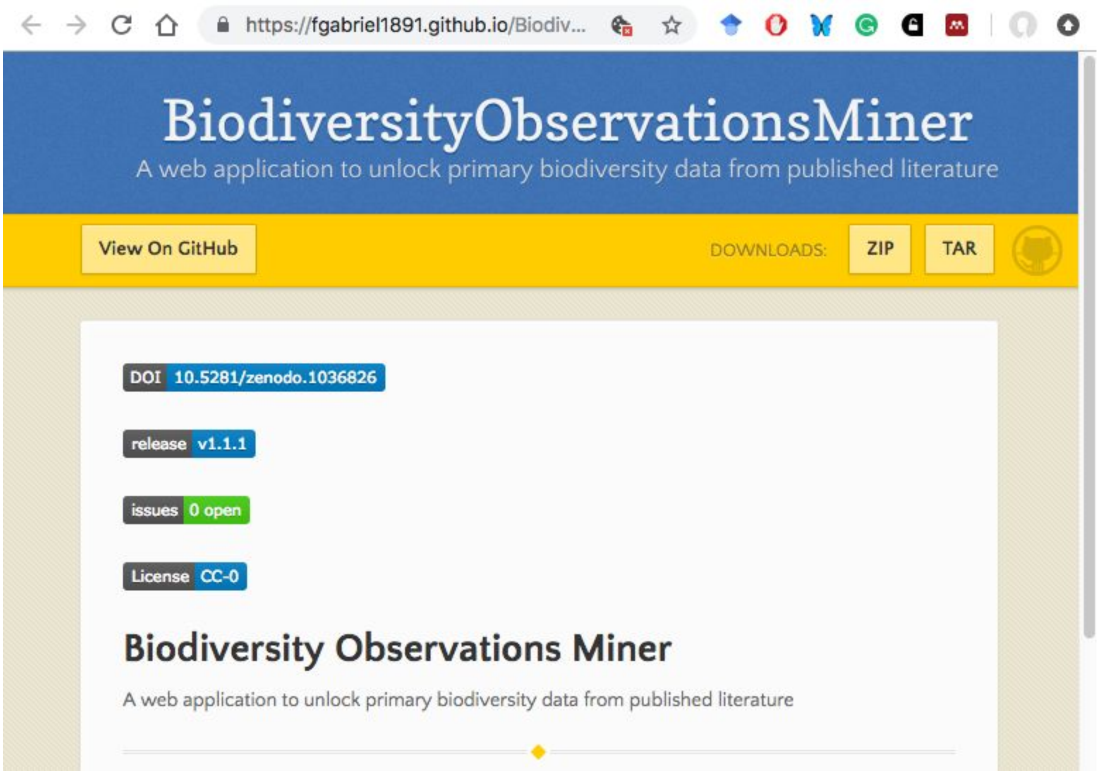

Figure 2: GitHub frontpage of Biodiversity Observations Miner. To download the application files click on the “ZIP” or “TAR” buttons. Save it into a local folder and de-compress the downloaded folder.

c) Open the R-project container file (the one with the R icon inside a cube), once within R Studio, open either the ui.R or the server.R file and click on the Run App button.

Important!: Be sure to have R (https://www.r-project.org/) and R studio (https://www.rstudio.com/) installed and running at your computer before opening this file

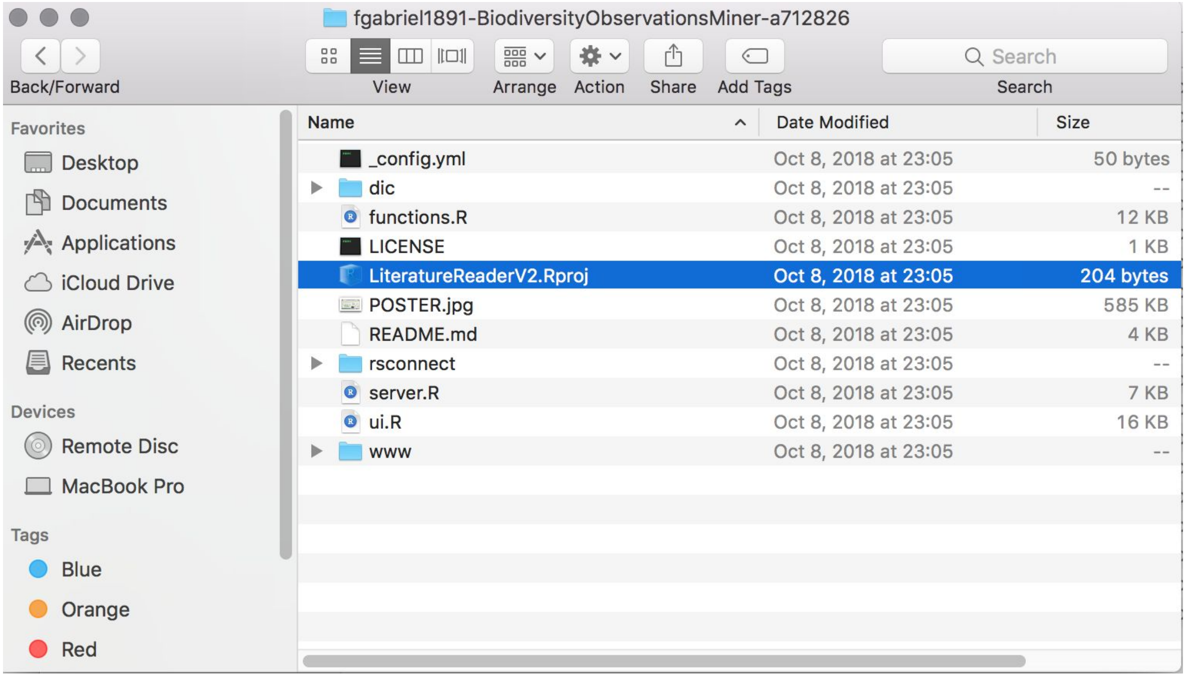

Figure 3: Contents of the Biodiversity Observations Miner repository folder. Open the R container file (selected in blue) to open the application in RStudio

2.3. Alternate way to run locally (for more advanced users)

In the R console in R studio run: **shiny::runGitHub("BiodiversityObservationsMiner", "fgabriel1891")**

Important!:
be sure to have installed the following R packages before running the application:
shiny, DT, stringi, taxize, tibble, udpipe, shinythemes, shinydashboard, reshape2
to install a package(s) type in the R console: ***install.packages(c("package1Name", "package2Name", "package3Name"))***

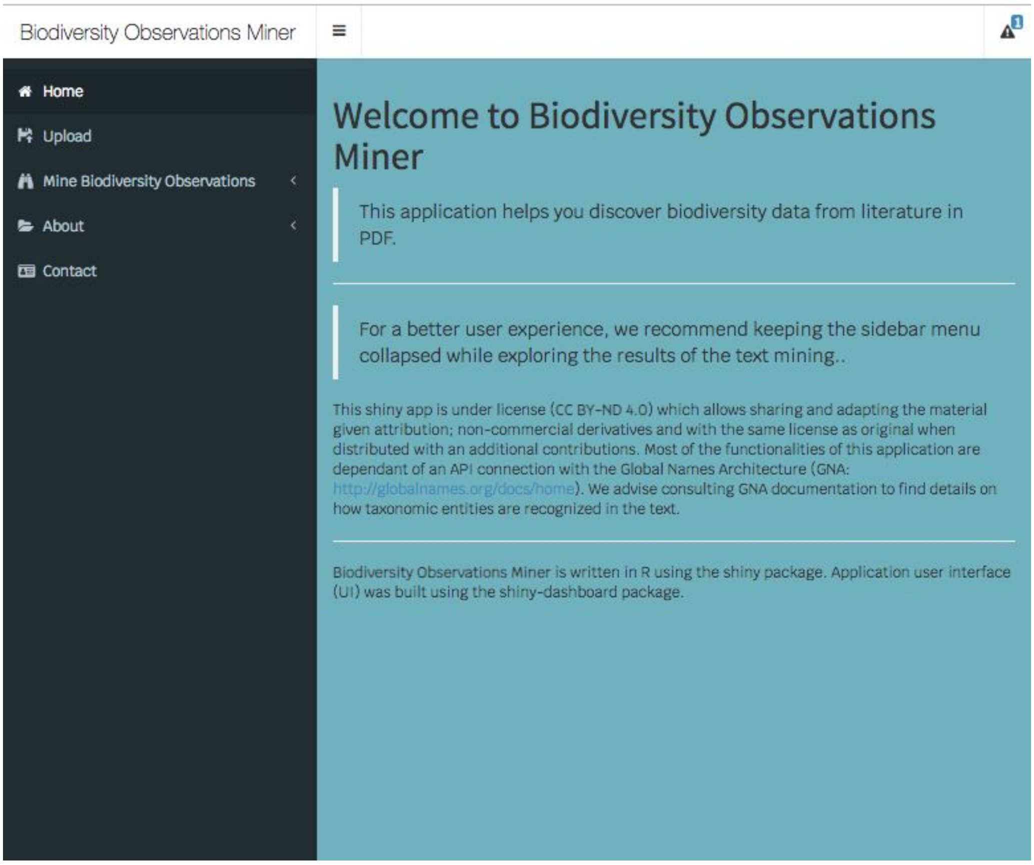

Figure 4: Biodiversity Observations Miner Homepage.

3.- Application structure

Currently, BOM have 5 tabs located in the sidebar (dark blue in Figure 4).

a) Home: Renders the homepage
b) Upload: Tab to upload the pdf files
c) Mine Biodiversity Observations: Tab to mine the literature uploaded in the Upload tab
d) About: Some extra information about the application
e) Contact: Contact information with the authors of BOM

In the header of the application (white bar in Figure 4), next to the title, there is a button to collapse the sidebar at will. This enhances the user experience once observing the results of the literature mining.

4.- Uploading literature to BOM

To upload the PDF files to be mine go to the Upload tab, here you will find a green box. Click on the Browse button and a window will open. Locate the folder with the PDF files that you prepare in step 1. Select all (or a subset) of the files within that folder and click accept. A blue bar in the green box will show you the progress of the upload. Once the files have been uploaded, proceed to the Mine Biodiversity Observations Tab.

Important!
Currently the application has a limit of max 30Mb for uploading files (this is to avoid users uploading large pdfs to the web server). However, if you need exceed the limit you can run the application in subsets or running BOM locally. To increase the file size limit when running locally, you can modify it in the line 27 of the server.R file.

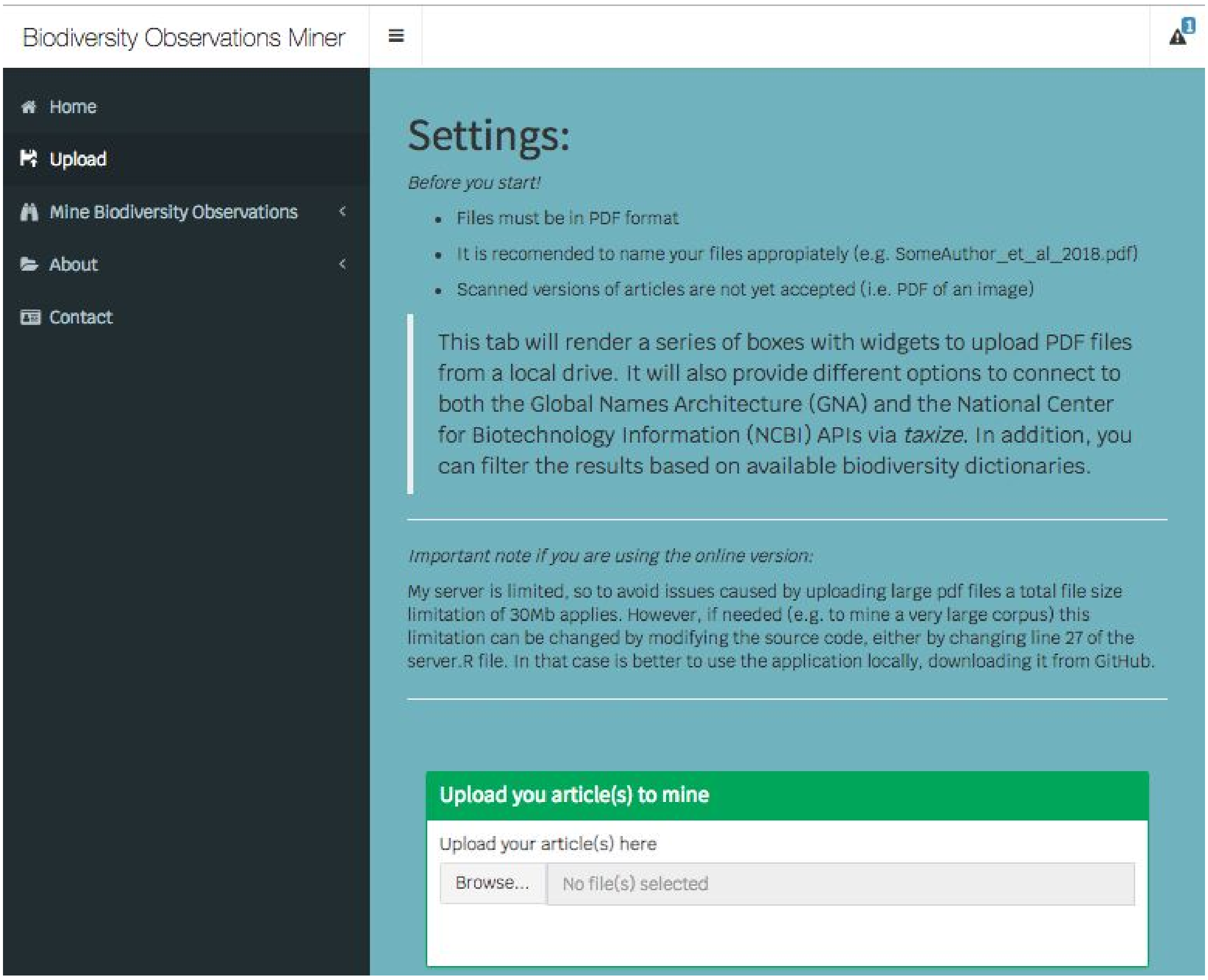

Figure 5: Upload tab of BOM. Clicking in the Browse button will open a window where the user can locate the file(s) to mine from its device.

5.- Mine Biodiversity Observations

By clicking on this tab, two subtabs will appear. **byTaxa** and **byKeywords**.

5.1. Using taxonomy to find Biodiversity Observations (byTaxa subtab).

In this subtab a series of steps need to be followed to retrieve snippets of text containing biodiversity observations.

a) Select taxonomic resolution of taxa mining.

BOM uses the Global Names Recognition and Discovery (GNA) API to perform the PDF Optical Character Recognition (OCR) and identify the taxa contained in the literature corpus.

The user can select to identify only the scientific names present in the literature (faster) or also connect to the ncbi database to identify the family and class of such names found (requires more time). In both cases, the action is triggered with the Get Taxa button. A progress bar in the bottom right corner of the application will allow the user to track the progress of the identification. Please be patient as this step can be time consuming especially if there is a large number of files and the user have selected to provide family and class information.

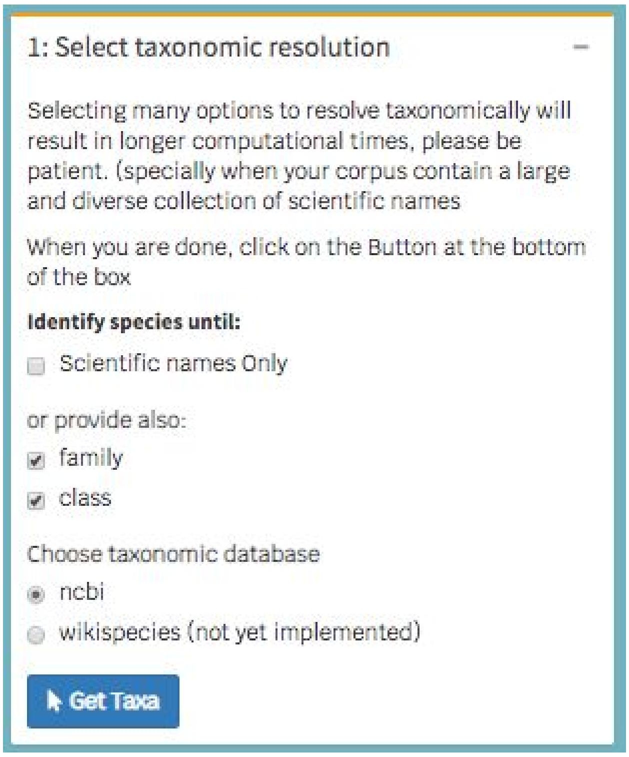

Figure 6: Taxonomic resolution settings inside the **byTaxa** subtab of BOM.

b) Select a taxa

As result from the previous step, a clickable datatable, containing the taxonomic information will render in the 2: Select a taxa box Below this box, in the top corner a message will appear with information whether all files were correctly mined or if there was some issue will any of the files provided. In the datatable the user can filter or search for a particular taxa. To obtain more information, click the row of a taxa of interest.

c) Infer context

Clicking the Infer Context button in the box 3: Render context will provide the word co-occurrences found for the text statements containing the taxa of interest selected in the previous step. This is useful to rapidly assess the content inside the text associated to that particular taxa.

d) Text snippets

In this box the user can find the text statements mentioning the taxa of interest in all the literature uploaded. The statements are classified by file. Moreover, the user can search/filter for a particular statement by typing a desired word from the previous step in one of the search boxes.

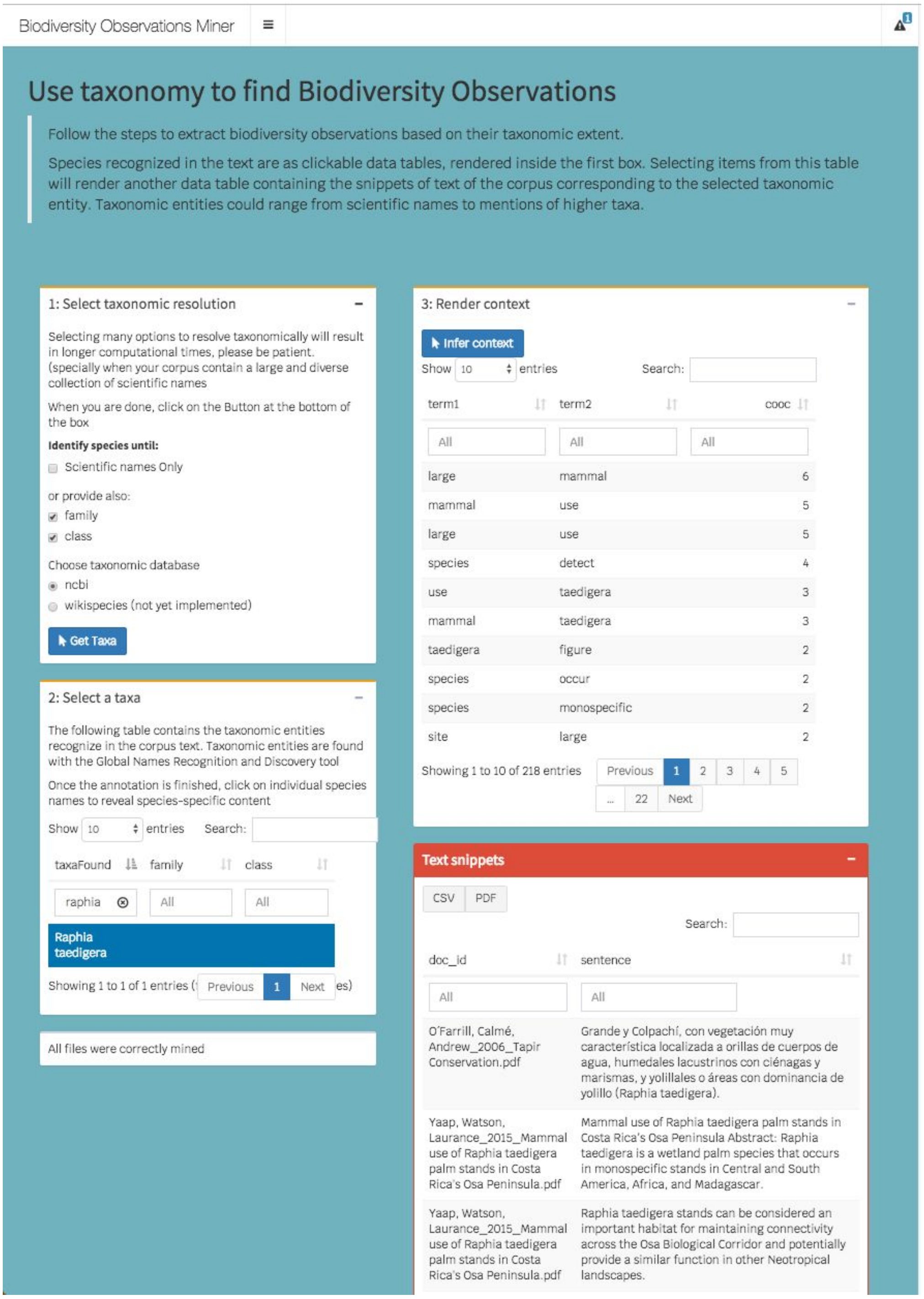

Figure 7: Finding text snippets associated to a particular taxa. The user selects the taxa of interest by clicking in the 2: Select a taxa box, find the context by the word co-occurrences in box 3: Render Context and can search for specific text snippets in the Text snippets box (red header).

5.2. Using word co-occurrences to find Biodiversity Observations (byKeywords subtab).

a) To annotate the literature corpus first click on the Find Word Associations button
b) (optional) To filter the results based on biodiversity dictionaries, mark the checkbox and select a biodiversity dictionary from the dropdown list
c) A clickable data-table will render with three columns: **term1**, **term2** and **cooc**. The first two contain the co-occurring terms and the third one provides information on the frequency of co-occurrence.
d) Explore and click a row and the corresponding text snippets will render in the box at the right (red header).

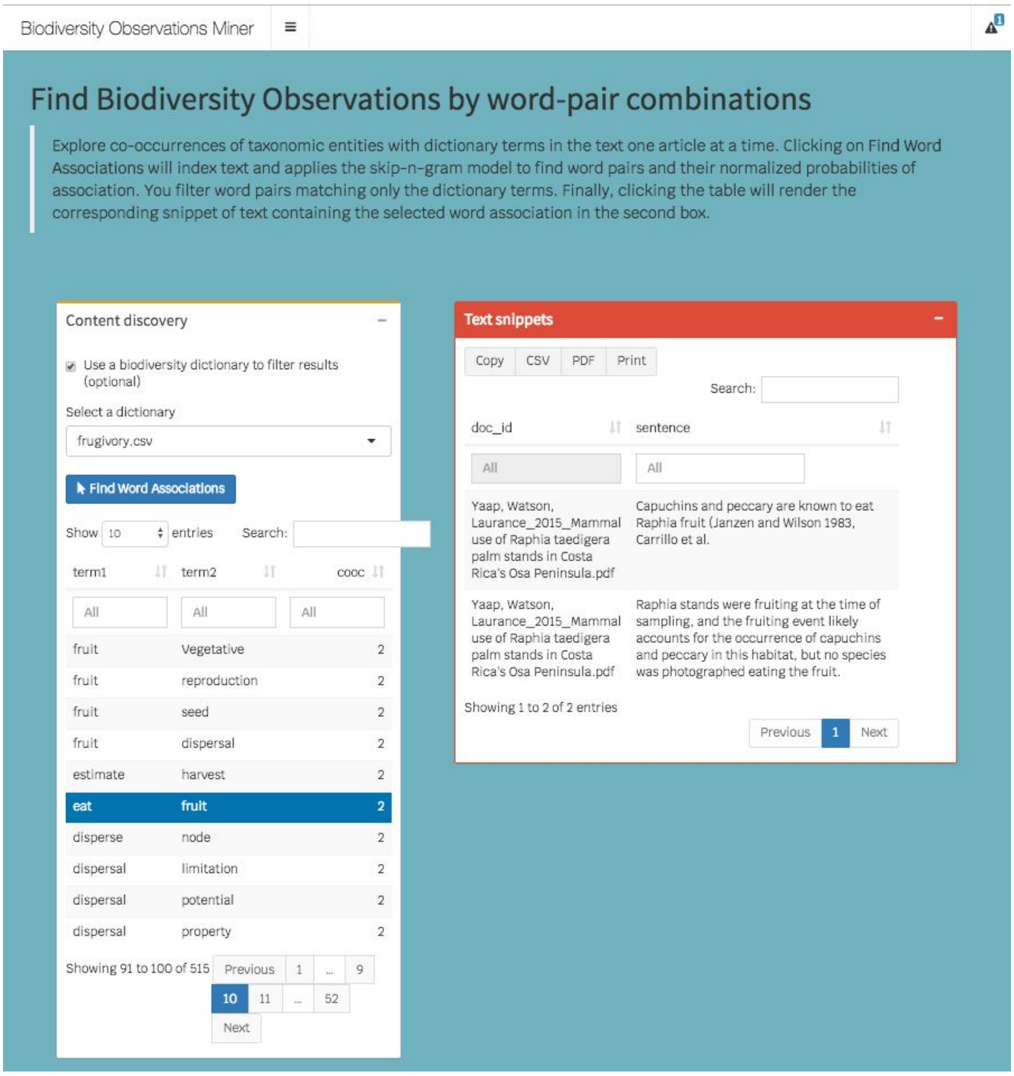

Figure 8: **byKeyworkds** sub tab. In this tab the user can find biodiversity information tagged by particular word co-occurrence combinations. There is the option to filter the word co-occurrence results by particular biodiversity dictionaries. Currently available biodiversity dictionaries include frugivory and pollination.

Important!
Available biodiversity dictionaries in BOM currently include frugivory and pollination. A biodiversity dictionary consist of a list of curated terms known to describe a particular observation in biodiversity. Such terms can be created by hand picking terms from text known to mention the biodiversity observation of interest. To add more biodiversity dictionaries into BOM please open a GitHub issue (https://github.com/fgabriel1891/BiodiversityObservationsMiner/issues/new) and provide us with a list of desired terms so we can included into the web server. If you want to add a biodiversity dictionary locally, prepare a .csv file with a custom list of terms as a 1 column where each term is on a different row. The header of the column should be named "dictionary". Place this file in the /dic folder in the repository. The dictionary now will be available in your version of BOM.

To report a bug, comments or questions please open a new GitHub issue!

https://github.com/fgabriel1891/BiodiversityObservationsMiner/issues/new

Contact:
Gabriel Muñoz
fgabriel1891@gmail.com
/fgabriel1891 (GitHub)
